# *In vitro* and *in vivo* Anti-leishmanial Potential of [Ag (PTA) _4_ ]BF _4_ and [Ag(HBPz _3_ )(PPh _3_ )] Silver Complexes

**DOI:** 10.1590/0037-8682-0478-2021

**Published:** 2022-04-08

**Authors:** Pauline de Faria Soldera, Ana Flavia da Silva Chagas, Anny Maisa Vargas Brasil, Claudia Dantas Comandolli-Wyrepkowski, Marina Porchia, Antonia Maria Ramos Franco Pereira

**Affiliations:** 1 Universidade Federal do Amazonas, Programa de Pós-Graduação Stricto Sensu em Inovação Farmacêutica, Manaus, AM, Brasil.; 2 Instituto Nacional de Pesquisas da Amazônia, Laboratório de Leishmaniose e Doença de Chagas, Manaus, AM, Brasil.; 3 Istituto di Chimica della Materia Condensata e di Tecnologie per l’Energia, Pádua, Italy.

**Keywords:** American Tegumentary Leishmaniasis, Antileishmanial Activity Metallic Complexes, Silver

## Abstract

**Background:**

American tegumentary leishmaniasis is a parasitic disease known for being difficult to treat; therefore, the search for more effective therapeutic methods is necessary. The objective of this study was to evaluate the *in vitro* and *in vivo* antileishmanial activity of silver complexes [Ag(PTA)_4_]BF_4_ (Ag1) and [Ag(HBPz_3_)(PPh_3_)] (Ag2) against *Leishmania* (*Leishmania*) *amazonensis* [*L*. (*L*.) *amazonensis*] and *Leishmania* (*Viannia*) *guyanensis*.

**Methods:**

*In vitro* bioassays were performed to evaluate the activity of the complexes against promastigote and amastigote forms and evaluate their cytotoxicity. *In vivo* experiments were performed with hamsters (*Mesocricetus auratus*) infected and treated topically with two gels containing each metallic complex.

**Results:**

Both complexes reduced the number of viable parasites against the promastigote forms of *L*. (*L*.) *amazonensis*. Ag2 was mainly effective against the amastigote forms. The Ag2 complex did not present cellular cytotoxicity, and regarding the selectivity index, both complexes were considered acceptable, with Ag2 having the best selectivity index in murine peritoneal macrophages in relation to *L. (L.) amazonensis.* Ag2 showed better results in the topical treatment against infections caused by *L*. (*L*.) *amazonensis*, with a small reduction in the lesion volume after the 14^th^ day of treatment and less parasitic load at the lesion site.

**Conclusions:**

Ag2 was more effective than Ag1 against *L*. (*L*.) *amazonensis*.

## INTRODUCTION

American tegumentary leishmaniasis (ATL) is a noncontagious infection caused by parasites of the genus *Leishmania*. The clinical forms in humans include cutaneous (CL), diffuse disseminated cutaneous (DDCL), and cutaneous mucosal (CML). The drugs used in the treatment belong to the class of pentavalent antimonials, meglumine antimoniate (Glucantime®), and sodium stibogluconate (Pentostam®), whose toxicity and treatment failure are common. Therefore, differentiated treatment using pentamidine isethionate (Pentamidine®), amphotericin B®, and paromomycin® may be adopted when a satisfactory response to antimonial treatment is not obtained^1^. 

Thus, the search for more effective therapeutic options for American tegumentary leishmaniasis (ALT) treatment has become evident, and an alternative is the use of metal complexes. The use of metals as drugs has considerable potential and can increase the arsenal of drugs available to treat diseases, particularly leishmaniasis^2^. 

Metal complexes consist of metal ions bound to atoms, ions, or molecules are called ligands. The interaction between a metal ion and different ligands determines the availability of a variety of coordination compounds, which display different metal oxidation states and increased stability. The nature of the ligand and its ability to form chelates with metals favor the biological activities of the complexes^3^. 

Related literature contains reports on the cytotoxic activity of silver-based complexes associated with phosphine-type ligands in tumor cells. These reports show that metal-phosphine compounds present lower cytotoxicity and indicate that the nature of the ligand is related to the activity of the complex^4^. Some studies have compared the activity of phosphine homoleptic silver complexes to the activity of mixed ligand (phosphine and trispyrazolylborate) complexes against different cancer cell lines endowed with different sensitivities to platinum drugs^5^. 

Based on reports regarding the use of silver in disease treatments, this study aimed to evaluate whether the metal complexes [Ag(PTA)_4_]BF_4_ and [Ag(HBPz_3_)(PPh_3_)] exhibit cytotoxic activity in murine and human cells. In addition, we also evaluated their in vitro activity against promastigote and amastigote forms of *Leishmania* (*Leishmania*) *amazonensis* and *Leishmania* (*Viannia*) *guyanensis*, as well as the therapeutic and pharmacological effects *of* topical treatment using two gels, each containing a distinct metal complex, in hamsters infected with these species.

## METHODS

### Chemical compounds

Two metal complexes, [Ag (PTA)_4_]BF_4_, [PTA = 1,3,5-triaza-7-prhophaadamantane] (Ag1) and [Ag(HBPz_3_)(PPh_3_)], [PPH_3_ = triphenylphosphine and [HB(pz)3] = tris(pyrazol-1-yl)borate] (Ag2), were used. Chemical synthesis of the compounds^4^ ^,^ ^5^ was performed using the Istituto di Chimica della Materia Condensata e di Tecnologie per l’Energia (ICMATE) and sent for biological study at the National Institute for Amazonian Research (INPA). This study was part of the Vaikutus Project Consortium (FP6-PEOPLE-IRES-2011-295262).

The samples of the complexes were diluted in dimethyl sulfoxide (DMSO) at a concentration of 2% and Roswell Park Memorial Institute **(**RPMI) medium and filtered through membranes of 22 µm under sterile conditions. The activity of the complexes was compared to that of Glucantime®.

### In vitro assays

### Cytotoxicity in murine peritoneal macrophages and human monocytes

Peritoneal macrophages were extracted from Balb/c (*Mus musculus*) mice, from colonies of the Central Vivarium at the INPA, with the approval of the Ethics Committee on the Use of Animals (CEUA - INPA 023/2020; A*ppendix A)*. The animals were anesthetized with ketamine hydrochloride (Dopalen®), and a ventral incision was made. The RPMI medium was introduced; and the abdominal massage was performed for macrophage activation, followed by aspiration, and centrifuged at 1,500 g for 10 min. Cell concentration was adjusted to 10^4^ macrophages/mL^6^. 

Human monocytes were obtained after approval from the Research Ethics Committee (CEP-UFAM: 29406319.2.0000.5020) (Appendix B). Blood samples were collected from all patients. Phosphate-buffered saline (PBS) 1 x was added, and the blood/PBS was added gently on Ficoll-Histopaque (Sigma-Aldrich®) [density of 1077] and centrifuged for 30 min/200 g^7^. Peripheral blood mononuclear cells were collected, added PBS 1 x, and centrifuged for 3 min at 400 × g. The cell concentration was adjusted to 10^4^ monocytes/mL^7^.

The assay was performed in 96-well plates (KASVI®). The RPMI medium was added to the cells at a concentration of 10^4^ cells/mL/well. The metal complexes were added at concentrations of 160, 80, 40, and 20 mol/mL in triplicate as a positive control, and Glucantime® was used at the same concentration as the metal complexes. The negative controls used DMSO at 4 concentrations (2%, 1%, 0.50%, and 0.25%), and the RMPI medium containing the cells remained exposed to the substance for up to 24, 48, and 72 h. 

After each period, a solution of [3-(4,5-dimethylthiazol-2yl) -2,5-diphenyltetrazolium] bromide (MTT) was added to the wells at (concentration of 0.5 mg/mL). The plates were incubated for 4 h at 37 °C and then subjected to spectrophotometry (Biotek®) at an absorbance of 590 nm. Three independent trials were performed with peritoneal macrophages and human monocytes.

### Parasites

We used the species *L.* (*Leishmania*) *amazonensis* [*L. (L.) amazonensis*] (MHOM/BR/2009/IM5584) and *L.* (*Viannia*) *guyanensis* [*L. (V.) guyanensis*] (MHOM/BR/1975/M4147), which had been cultivated in the biphasic medium Novy - MacNeal - Nicolle (NNN) and amplified in the RPMI liquid medium (Himedia®). The culture was centrifuged for 15 min at 4,400 g. Parasites were adjusted to a concentration of 10^4^ parasites/mL.

### Assays with promastigote forms

The parasites were used at a concentration of 10^4^ parasites/mL. The assay was performed in 96-well plates (KASVI®), and then the same procedure and concentrations of the substances were used in the cytotoxicity assay, with negative control and culture medium containing parasites. The activity of the complexes was evaluated at 24, 48, and 72 h intervals using a Neubauer chamber. In addition, cellular viability was evaluated using the assay. Three independent tests were conducted.

### Assays with amastigote forms

The assay against amastigote forms was performed using murine peritoneal macrophages and human monocytes, obtained as described for the cytotoxicity assay. The cells were incubated for 48 h in a 24 well-plate containing glass coverslips with the RPMI medium, and cell volume adjusted to 10^5^ macrophages/monocyte/mL.

The adhered cells were infected with promastigote forms of *Leishmania* spp. and left for 2 h. The test substances were then added at the concentrations used in previous trials and exposed for 24, 48, and 72 h. Coverslips were stained using a Panoptic kit (Laborclin® - Laborclin Products for Laboratories Ltd, Pinhais, Brazil) and quantified using optical microscopy at 400 × magnification. A total of 100 cells were quantified to evaluate the number of infected and uninfected macrophages and internalized amastigotes. Three independent assays were performed on peritoneal macrophages and human monocytes.

### Selectivity index (SI)

The SI is the relationship between the cytotoxic activity of the tested compound and its anti-leishmanial activity. In our study, the SI was calculated from the ratio of cytotoxicity for macrophages/monocytes with a cytotoxic concentration at 50% (CC_50_) and action against amastigote forms in each of these cell types with an inhibitory concentration at 50% (IC_50_), using the equation: $SI\;=\;{CC}_{50}{IC}_{50}-{AMA}^{3}$

### Statistical analysis

Statistical analysis was performed using linear regression to calculate IC_50_ using GraphPad Prism® (GraphPad Software Inc. version 6.0, for Windows). Results are expressed as mean ± standard deviation.

### Preclinical study

### Preparation of the gel

To prepare the Carbopol®-based gel, Nipagin® was mixed with distilled water until its total solubilization, and then Carpobol® was added. Then, the metal complex was added and incorporated separately in each gel, and triethonolamine was added gradually until a thick gel was obtained^8^. Finally, the metal complexes were mixed at a concentration of 3 mg/L, a value defined for comparative purposes with the positive control of the study (Glucantime®) administered at the same concentration via the intramuscular (IM) route. 

### Experimental animals

For the assays, 72 adult male aged 60-days golden hamsters (*Mesocricetus auratus*) that were free of pathogens and had been previously obtained from the Central Vivarium at the INPA (CEUA 060/2018) were used [A*ppendix C]*. The animals were housed in stainless steel cages in air-conditioned rooms, with controlled light, temperature (22-25 °C), humidity (50-60%), and food and water *ad libitum*.

### Infection and treatment of animals

Animals were separated into six groups. Five groups were infected with 100 µL of a solution containing 10^6^ promastigote/mL of *Leishmania* spp. The first experiment involved 36 animals infected with *L*. (*L*.) *amazonensis,* and the second experiment involved 36 animals infected with *L*. (*V.*) *guyanensis*. Animals were divided into the following groups:

- Group I: Uninfected control
- Group II: Untreated, infected control
- Group III: Infected and treated with Glucantime® (IM)
- Group IV: Infected and treated with Ag1 (lesions treated topically once a day)
- Group V: Infected and treated with Ag2 (lesions treated topically once a day)
- Group VI: Infected and treated with gel without the addition of complexes (lesions treated topically once a day)

To evaluate the course of infection, the total volume of the lesion (length, width, and height) was measured using a caliper (Zaas® Precision). Animals were weighed once per week. After 30 days of treatment, the animals were euthanized using ketamine hydrochloride (Dopalen®) combined with xylazine hydrochloride (Anasedan®) following the Animal Use Ethics Commission (CEUA) protocols (060/2018).

### Evaluation of topical treatment

For parasitological studies, a sample of the tissue from the lesion region lesion in each animal was sectioned, and the fragment was printed on a glass slide and stained using the Panoptic kit (Laborclin®). The slides were evaluated using optical microscopy, 30 fields were counted, and the analysis and quantification of infected and uninfected cells and the quantity of internalized amastigote forms were performed. Fragments of each animal’s skin lesions and liver were also sectioned and cultured in an NNN medium for 7 days. After this period, an aliquot containing the fragments was evaluated using optical microscopy for parasitological analysis.

### Statistical analysis

For the *in vivo* results, we used two-way analysis of variance to evaluate the statistical significance between the groups, followed by the Tukey test to compare the means of the groups at a 95% confidence interval. 

## RESULTS

The values of the *in vitro* results for the analysis of IC_50_ were defined as follows: <10 μM, highly active; 10-30 μM active; 30-50 μM moderately active; and >60 μM, not active. The parameters for determining these values were based on the results obtained in the literature^3^ ^,^ ^9^.

According to the IC_50_ values obtained against promastigotes of *L*. (*L*.) *amazonensis*, Ag1 and Ag2 were considered highly active, with statistical differences when compared to Glucantime® (IC_50_ <10 and 292.14). In contrast to *L*. (*V.*) *guyanensis,* the complexes presented IC_50_ values that were considered inactive according to the criteria established in the study (Table 1).

**TABLE 1:** IC_50_ values of the activity of silver complexes against promastigote forms of *Leishmania (Leishmania) amazonensis* and *Leishmania (Viannia) guyanensis*.

	IC_50_ values (μM (10^-6^ M) ± Standard Deviation)/Incubation period Leishmania (Leishmania) amazonensis (IM5584)		
Compounds	24 h	48 h	72 h
Ag1	<10**	69.18 ± 3.03	104.50 ± 1.47
Ag2	<10**	<10**	54.33 ± 0.76
Glucantime®	292.14 ± 2.40*	343.10 ± 2.37*	435.60 ± 3.03*
	**IC**_50_ **values (μM (10** ^-6^ **M) ± Standard Deviation)/Incubation period** **Leishmania (Viannia) guyanensis (M4147)**		
**Compounds**	**24 h**	**48 h**	**72 h**
Ag1	75.14 ± 0.53	130.30 ± 2.94	228.90 ± 1.26
Ag2	108.51 ± 0.54	120.81 ± 2.04	150.30 ± 2.75
Glucantime®	286.60 ± 1.28	376.51 ± 2.74	453.70 ± 3.18

**IC**_50_ **:** Inhibitory concentration at 50% (μMol (10^-6^ mol) ± standard deviation). *P*<0.05 Significant differences, according to the Tukey test. *Values marked with asterisks indicate that the compound is statistically different from the other compounds. **Values marked with an asterisk means that the compound are statistically similar to each other.

When evaluated in relation to cytotoxicity in murine peritoneal macrophages and human monocytes, Ag1 was toxic to both cell types. However, when administered to peritoneal macrophages, the cells presented lower cell viability (IC_50_ 52.66) in human monocytes (IC_50_ 69.37) and presented statistical differences when compared with Glucantime® (IC_50_ 178.99 and 398.40), indicating that this complex may affect cell integrity. 

Ag2 did not show cytotoxic activity against any of the cell types evaluated. However, it did present viability in relation to treatment periods in peritoneal macrophages (IC_50_ 209.44), indicating that prolonged exposure to the complex did not interfere with cell viability. Furthermore, the same result was obtained when tested on human monocytes (IC_50_ 436.80), indicating the possibility of cells presenting greater viability after longer exposure to the complex (Table 2).

**TABLE 2:** Cytotoxic activity in murine peritoneal macrophages and human monocytes with CC_50_ values of silver complexes.

CC_50_ values (μM (10^-6^ M) ± Standard Deviation)/Incubation period - peritoneal macrophages					
Compounds	24 h	48 h	72 h
Ag1	52.66 ± 1.87*	95.49 ± 1.13*	92.62 ± 2.63*
Ag2	200.88 ± 0.20**	206.38 ± 1.14**	209.44 ± 2.00**
Glucantime®	178.99 ± 0.23	205.21 ± 1.25**	211.61 ± 2.48**
**CC**_50_ **values (μM (10** ^-6^ **M) ± Standard Deviation)/Incubation period - human monocytes**					
**Compounds**	**24 h**	**48 h**	**72 h**
Ag1	98.02 ± 0.99	94.30 ± 2.90*	69.37 ± 1.28
Ag2	175.19 ± 1.78	391.63 ± 2.47**	436.80 ± 2.75**
Glucantime®	149.36 ± 1.52	254.90 ± 2.74**	398.40 ± 1.18**

**CC**_50_ **:** 50% cytotoxic concentration (μMol (10^-6^ Mol) ± standard deviation). *P*<0.05 Significant differences, according to the Tukey test. * Values marked with asterisks indicate that the compound is statistically different from the other compounds; **Values marked with an asterisk means that the compound are statistically similar to each other.

The complexes' activity against the amastigote of *L*. (*L*.) *amazonensis* internalized in murine peritoneal macrophages showed that Ag1 caused a greater reduction of the infection in all periods than Ag2 and Glucantime®. Furthermore, in human monocytes, the same complex reduced infection (IC_50_ 23.28). 

The results with macrophages infected by the amastigotes of *L*. (*V*.) *guyanensis*, show that Ag2 presented a more significant reduction among the substances tested (IC_50_ 30.50). However, there was no significant difference between the complexes and Glucantime® in relation to the activity in infected monocytes (Table 3). 

The SI was calculated to evaluate the selectivity of the metal complexes in relation to host cells, macrophages, monocytes, and amastigote forms of the parasite. SI values greater than or equal to 10 were considered satisfactory^3^. The results of SI showed efficiency with peritoneal macrophages only when using Ag2 (Table 3).

**TABLE 3:** IC_50_ values of the activity of silver complexes against amastigote forms of *Leishmania (Leishmaia) amazonensis* and *Leishamania (Viannia) guyanensis*, and (SI) in peritoneal macrophages.

Complex	Period (h)	Amastigote IC_50_ (μM (10^-6^ M) ± standard deviation) Macrophages		Amastigote IC_50_ (μM (10^-6^ M) ± standard deviation) Monocytes		Selectivity index Macrophages	
		*L. (L.) amazonensis*	*L. (V.) guyanensis*	*L. (L.) amazonensis*	*L. (V.) guyanensis*	*L. (L.) amazonensis*	*L. (V.) guyanensis*
**Ag1**	24	6.09 ± 0.47	90.51 ± 0.70	23.28 ± 1.04	109.20 ± 0.63		
	48	14.91 ± 0.41	105.12 ± 0.42	69.04 ± 0.34	186.60 ± 0.57	3.89	3.64
	72	23.80 ± 1.51	116.25 ± 1.58	150.70 ± 0.91	270.30 ± 0.25		
Ag2	24	90.45 ± 1.33	65.44 ± 0.50	91.30 ± 0.9	237.10 ± 0.94		
	48	103.70 ± 1.59	30.50 ± 0.70	129.87 ± 0.23	235.70 ± 1.03	10.28	6.70
	72	112.87 ± 1.18	103.28 ± 1.17	230.10 ± 2.01	200.90 ± 0.90		
	24	57.08 ± 0.77	48.19 ± 0.77	294.60 ± 0.38	374.70 ± 0.64		
Glucantime®	48	279.20 ± 1.95	70.19 ± 1.62	317.20 ± 1.75	372.60 ± 1.75	-	-
	72	318.67 ± 1.48	101.06 ± 1.02	391.00 ± 1.38	427.80 ± 1.32		

**IC**_50_ **:** Inhibitory concentration at 5 m% (μMol (10^-6^ Mol) ± standard deviation); **SI:** Selectivity index; *P*<0.05 Significant differences, according to the Tukey test.

In the experimental animals, the analysis of the topical treatment against lesions caused by *Leishmania* spp. was performed by measuring the total volume of the lesion and quantifying the infected cells and internalized amastigotes in the lesion tissue. In addition, animals treated with the metal complex were compared with the group of animals treated with Glucantime® and with animals that received no treatment. 

**FIGURE 1:**
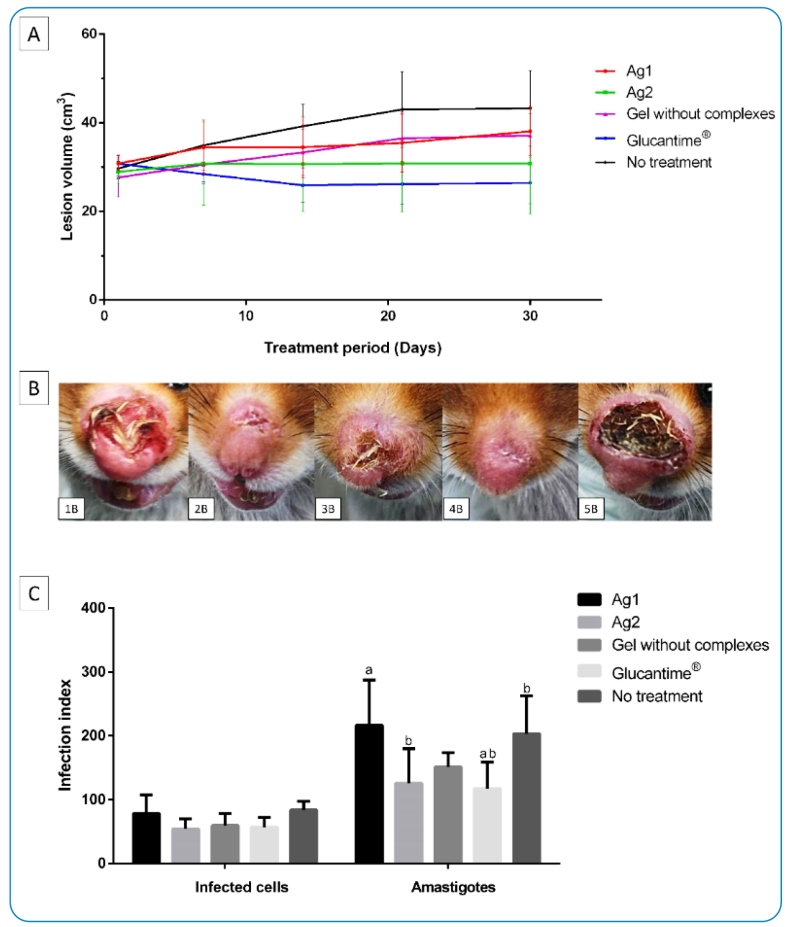
**(A)** Effect of topical treatment of lesions in *M. auratus* infected with *L*. (*L*.) *amazonensis* using 0.3 mg/day of gel with metal complexes and the group treated with gel without addition of the complexes. Positive control animals treated with Glucantime® IM 3 mg (Sb^5^)/kg/day^1^. Negative control: animals without treatment. The data represents the mean between the groups. **(B)** macroscopic clinical appearance of lesions in *M. auraus* infected in the nose after 30 days of treatment. **(1B)** animal treated with gel containing Ag1; **(2B)** animal treated with gel containing Ag2, **(3B)** animal treated with gel without addition of the complexes, **(4B)** animal treated with Glucantime® IM 3 mg (Sb^5^)/kg/day^1^_,_ **(5B)** untreated infected animal (six animals/group). (C) Infection index and amastigotes internalized in cells and quantified in lesions. Data represent the means. Letters indicate statistical differences according to the Tukey’s test (*P*>0.05). **IM:** intramuscular.

In the treatment of lesions caused by *L*. (*L*.) *amazonensis* (Figure 1), after the 14^th^ day of treatment, there was a reduction in lesion volume in animals treated with Ag2, and no statistical difference was observed compared with the drug Glucantime® (mean difference of 4.38). Furthermore, there was no significant difference in the weights of the animals between the groups.

Edema, local ulcerative processes, morphological features, and evolution of lesions caused by *L*. (*L*.) *amazonensis* were observed. The animals treated with Glucantime® showed a reduction in lesion volume; however, no clinical cure was obtained. The animals treated with Ag1 and the gel without adding the complexes showed an evolution of lesions. Statistical differences were observed only in relation to the infected and untreated animals and other groups (Figure 1). 

The values for infected cells present in lesion fragments demonstrate that Ag1 presented a greater number of internalized amastigotes, with a statistical difference compared to Glucantime®. In addition, the group treated with Ag2 showed a statistically significant difference compared to the group without treatment (Figure 1). 

The parasitological diagnosis was performed using cultures of the lesion fragments of each animal in the NNN medium to confirm the infection. Viable parasites were observed in all lesion fragments, including those treated with Glucantime®. 

**FIGURE 2:**
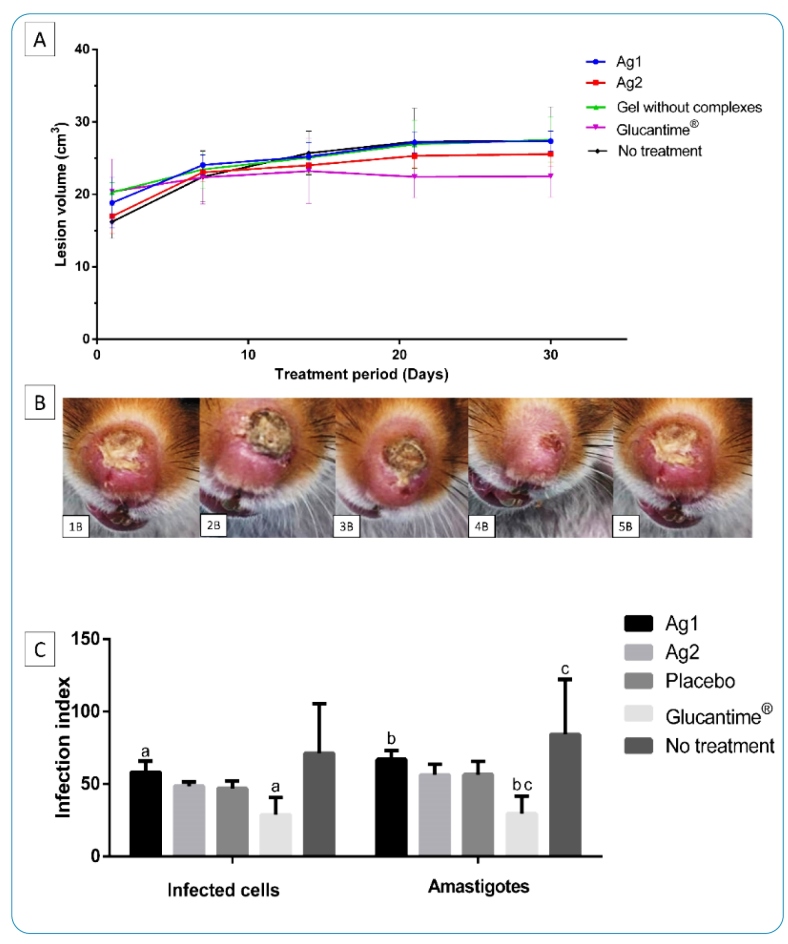
**(A)** Effect of topical treatment of lesions in *M. auratus* infected with *L*. (*V*.) *guyanensis* using 0.3 mg/day of gel with metal complexes and treated group with gel without addition of the complex. Positive control animals treated with Glucantime® IM 3 mg (Sb^5^)/kg/day^1^. Negative control: animals without treatment. The data represents the mean between the groups. **(B)** macroscopic clinical appearance of lesions in *M. auraus* infected in the nose after 30 days of treatment. **(1B)** animal treated with gel containing Ag1; **(2B)** animal treated with gel containing Ag2, **(3B)** animal treated with gel without addition of the complexes, **(4B)** animal treated with Glucantime® IM 3 mg (Sb^5^)/kg/day^1^_,_ **(5B)** untreated infected animal (six animals/group). **(C)** Infection index and amastigotes internalized in cells and quantified in lesions. Data represent the means. Letters indicate statistical differences according to the Tukey’s test (*P*>0.05). **IM:** intramuscular.

Figure 2 presents the results of the topical treatment of lesions caused by *L*. (*V.*) *guyanensis*. When treated with the gels, the lesions did not show a reduction in volume and demonstrated statistical differences compared to the group treated with Glucantime® after the 14^th^ day of treatment. The morphological aspects and evolution of the lesion volume were small. All animals showed a clinical cure. The groups treated with gels showed the evolution of the lesion. There was no significant difference in the weights of the animals between the groups.

In relation to the number of infected cells present in lesion fragments, the group treated with Ag1 presented the largest number of infected cells, with a statistical difference compared to the group treated with Glucantime®. Regarding the amount number of amastigotes internalized, the group treated with Ag1 presented the highest number compared to the treated groups, with a statistical difference compared to the group treated with Glucantime® (Figure 2). 

The parasitological diagnosis was performed using cultures of lesion fragments. The liver in the NNN medium confirmed the infection of all animals, with the presence of viable parasites in all lesion fragments, including the group treated with Glucantime®. 

## DISCUSSION

Much research has been conducted regarding new compounds that can act as therapeutic agents and present greater toxic activity to the parasite, with less damage to the cells of the human host. Among these compounds, metal complexes appear to be a more effective therapeutic arsenal for treating ATL, and silver has relevant medicinal properties due to its low toxicity to humans^9^.

The activity of a metal-based drug depends not only on the nature of the metal but also on the physicochemical properties of the ligands, determining the steric hindrance, lipophilicity, and total load of the resulting complexes, which, in many cases, are also able to interact with distinct biological targets. Therefore, it is not easy to compare the data obtained with different complexes because their activities can be directed to different biological targets and determine different mechanisms of action^3^.

The study of metallic complexes based on silver is still little explored, mainly because of the diverse nature of the ligands and treatment of pathologies associated with parasites, especially cutaneous leishmaniasis. 

Regarding cytotoxic activity, Ag1 presented a low CC_50_ when expressed on peritoneal macrophages, indicating the toxic activity of the cells. Articles^10^ ^,^ ^11^ have reported the cytotoxic activity of silver-based metal complexes *in vitro.* Cytotoxic activity was observed at concentrations above 1.5 ppm in macrophages and above 30 ppm in human monocytes. 

Studies have shown that the cytotoxic action of a metal complex is related to the activity of the target ligand^3^. Thus, the cytotoxic activity of Ag1 is directly linked to the type of coordinated phosphine, PTA, and nature of the metal. Other studies^4^ state that phosphine complexes associated with gold or silver could decrease the action of thioredoxin reductase protein (TrxR) by 50% at nanomolar concentrations, and this activity may be responsible for its cytotoxic activity in tumor cells. 

Regarding cytotoxicity, one study^4^ further described that by acting as an inhibitor of TrxR, silver and gold complexes with PTA ligands end up culminating in an alteration of the redox state of the cell, which leads to an overproduction of hydrogen peroxide and the oxidation of the Trx system, leading to conditions of cellular apoptosis, and consequently the cytotoxic action of the complex. 

The ability of a series of Ag complexes to selectively inhibit mammalian TrxR in the low nanomolar range was recently confirmed. Ag2 was a very strong inhibitor of both the purified enzyme and cell extracts and exerted remarkable cytotoxic activity toward a large panel of cancer cell lines. Interestingly, Ag2, together with TrxR inhibition and reactive oxygen species (ROS) overproduction, led to apoptotic cell death and displayed a marked ability to damage deoxyribonucleic acid (DNA)This dual mechanism is related to the HB(pz)_3_ ligand that interacts with DNA^5^. 

These results confirmed the key role of the nature of the ligands and their combinations, which may influence the complexes' cytotoxic activity, indicating the hydrophilic-lipophilic balance of the final Ag(I) complexes and their biological mechanism of action^5^.

One study^9^ tested silver compounds with N, n-diimine, and thiourea ligands against the promastigote forms of *L*. (*L*.) *amazonensis* (IC_50­_ 5.68-9.87 µm). This level of activity was also observed in our study for this species (IC_50_ <10) when subjected to the action of Ag2, corroborating the literature on the nature of the ligand in influencing the activity of the complex. Regarding the activity of silver-based complexes against *Leishmania,* a silver compound was reported, and the most effective was the dimeric species against promastigote forms of *L*. (*L*.) *amazonensis*, comparable to amphotericin B®. However, the functions of silver complexes have not yet been reported^3^. 

Against the promastigote forms of *L*. (*V.*) *guyanensis*, our complexes did not show satisfactory results; however, it is an innovative study because, to our knowledge, there are no other studies on this species using metal complexes. 

Our results against the amastigote of *L*. (*L*.) *amazonensis* in peritoneal macrophages, and human monocytes are in accordance with those reported by other authors who evaluated the activity of silver-based metallic complexes. They obtained an IC_50_ of 2.31 µg/mL and 1.30 µg/mL, in comparison with amphotericin B® (IC_50_ 1.20 µg/mL)^9^.

Reports in the literature using metal complexes against the amastigote forms of *L*. (*V.*) *guyanensis* are very rare. Only^12^ one study reported the use of gold complexes and observed moderate activity against amastigote forms of *L*. (*V.*) *guyanensis* internalized in macrophages (IC_50_ 3.5 µmol) values better than those found in our study (IC_50_ 30.50) using the same cell type.

*In vivo* studies using metallic complexes, mainly silver-based and topically applied, on lesions caused by *L*. (*L*.) *amazonensis* and *L*. (*V.*) *guyanensis*, were not found in our searches. Among the *in vivo* studies^13^ that evaluated the action of the ruthenium nitrosyl complex against *L*. (*V*.) *braziliensis*, a concentration of 300 µg/kg/day was administered by gavage. The results showed a reduction (51%) in lesion size.

The method of treatment with metallic complexes incorporated into gels was not found in the literature, which limits our comparison of the results. The decrease in the total volume of the lesions in animals caused by *L. (L.) amazonensis* after 14 days of treatment is presented as a precursor study, although a clinical cure has not been obtained.

Based on the results, Ag2 showed significant results, and it may be the target of a future study in relation to the treatment of integumentary leishmaniasis. The results of *in vitro* studies against *L. (L.) amazonensis* showed that it is active, and its cytotoxicity to murine peritoneal macrophages and human monocytes is low. In the topical treatment of animals infected with *L. (L.) amazonensis*, the results obtained from treatment with the gel containing Ag2 in its composition may present promising results.

## References

[1] Da Luz JSB, Oliveira EB, Martins MCB, Silva NH, Alves LC, Santos FAB (2015). Ultrastructural Analysis of Leishmania infantum chagasi Promastigotes Forms Treated In Vitro with Usnic Acid. ScientificWorldJournal.

[2] Barreiro E, Casas JS, Couce MD, Sánches A, Sordo J, Vázquez-Lópes EM (2014). Heteronuclear gold (I)-silver(I) sulfanylcarboxylates: synthesis, structure and cytotoxic activity against cancer cell lines. J Inor Biochemistry.

[3] Espuri PF, Reis LL, Peloso EF, Gontijo VS, Colombo FA, Nunes JB (2019). Synthesis and evaluation of the antileishmanial activity of silver compounds containing imidazolidine-2-thione. J Biol Inor Chem.

[4] Santini C, Pellei M, Papini G, Morresi B, Galassi R, Ricci S (2011). In vitro antitumor activity of water-soluble Cu (I), Ag (I) and Au (I) complexes supported by hydrophilic alkyl phosphine ligants. J Inorg Biochem.

[5] Dammak K, Porchia M, De Franco M, Zancato M, Naïli H, Gandin V (2020). Antiproliferative Homoleptic and Heteroleptic Phosphino Silver(I) Complexes: effect of Ligand Combination on their Biological Mechanism of Action. Molecules.

[6] Sasada M, Pabst MJ, Johnston RBJR (1983). Activation of mouse peritoneal macrophages by lipopolysaccharide alters the kinetic parameters of the superoxide-producing NADPH oxidase. J Biol Chem.

[7] Corkum CP, Ings PD, Burgess C, Karwowska S, Kroll W, Michalak TI (2015). Immune cell subsets and their gene expression profiles from human PBMC isolated by Vacutainer Cell Preparation Tube (CTP) and standard density gradient. BMC Immunol.

[8] Ferreira AO (2006). Formas farmacêuticas semi-sólidas.

[9] Segura DF, Netto AVG, Frem RCG, Mauro AE, Silva PB, Fernandes JA (2014). Synthesis and biological evaluation of ternary silver compounds bearing N,N-chelating ligands and thiourea: X-ray structure of [{Ag(bpy)(μ-tu)}2](NO3)2 (bpy = 2,2′-bipyridine; tu = thiourea). Polyhedron.

[10] Greulich C, Braum D, Peetsch A, Diendor J, Siebers B, Epple M (2012). The toxic effect of silver ions and silver nanoparticles towards bacteria and human cell occur in the same concentration range. RSC Advances.

[11] Mijnendonckx K, Leys N, Mahillon J, Silver S, Houdt RV (2013). Antimicrobial silver: uses, toxicity and potential for resistance. Biometals.

[12] Chaves JDS, Tunes LG, Franco CHJ, Francisco TM, Corrêa CC, Murta SMF (2017). Novel gold (I) complexes with 5-phenyl-1,3,4-oxadiazole-2-thione and phosphine as potential anticancer and antileishmanial agents. Eur J Med Chem.

[13] Nascimento NRF, Aguiar FLN, Santos CF, Costa AML, Hardoim DJ, Calabrese JS (2019). In vitro and in vivo leishmanicidal activity of a ruthenium nitrosyl complexes against Leishmania (Viannia) braziliensis. Acta Trop.

